# Novel Pestivirus Species in Pigs, Austria, 2015

**DOI:** 10.3201/eid2307.170163

**Published:** 2017-07

**Authors:** Benjamin Lamp, Lukas Schwarz, Sandra Högler, Christiane Riedel, Leonie Sinn, Barbara Rebel-Bauder, Herbert Weissenböck, Andrea Ladinig, Till Rümenapf

**Affiliations:** University of Veterinary Medicine, Vienna, Austria

**Keywords:** Flaviviridae, pestivirus, atypical porcine pestivirus, Bungowannah virus, congenital tremor, classical swine fever virus, Linda virus, viruses, Austria, pigs, livestock

## Abstract

A novel pestivirus species was discovered in a piglet-producing farm in Austria during virologic examinations of congenital tremor cases. The emergence of this novel pestivirus species, provisionally termed Linda virus, in domestic pigs may have implications for classical swine fever virus surveillance and porcine health management.

The genus *Pestivirus* consists of 4 approved species within the family *Flaviviridae* (*1*). Besides bovine viral diarrhea virus 1 (BVDV-1), BVDV-2, border disease virus (BDV), and classical swine fever virus (CSFV), several unassigned strains and tentative species are represented by the so-called atypical pestivirus strains. An atypical pestivirus of swine emerged in 2003 in a commercial pig-breeding farm in Australia and was later termed Bungowannah virus (BV). This well-studied virus caused reproductive disorders, stillbirth, and sudden death in piglets, resulting in the loss of ≈50,000 animals in the 2 affected farms. Because of its marked pathogenicity, BV was considered a threat to global pig health, but this virus or relatives were never found at other locations (*2*).

Recently, a novel group of porcine pestiviruses, termed atypical porcine pestiviruses (APPVs), was discovered. These viruses were identified in North America (*3*,*4*) and subsequently detected in Europe (*5*–*8*). There is strong evidence that APPVs are a causative agent behind the type A-II congenital tremor (CT A-II) syndrome of piglets (*4*). CT A-II is characterized by a generalized shaking of the whole body associated with variable degrees of hypomyelination in the brain and spinal cord. However, hypomyelination is also apparent in the brains of fetuses of sheep, cattle, and pigs after late-gestation state infection with BDV, BVDV, or CSFV (*9*). We report results of an investigation of CT in piglets on a farm in southeastern Austria in which a novel pestivirus species was discovered.

## The Study

**Figure 1:**
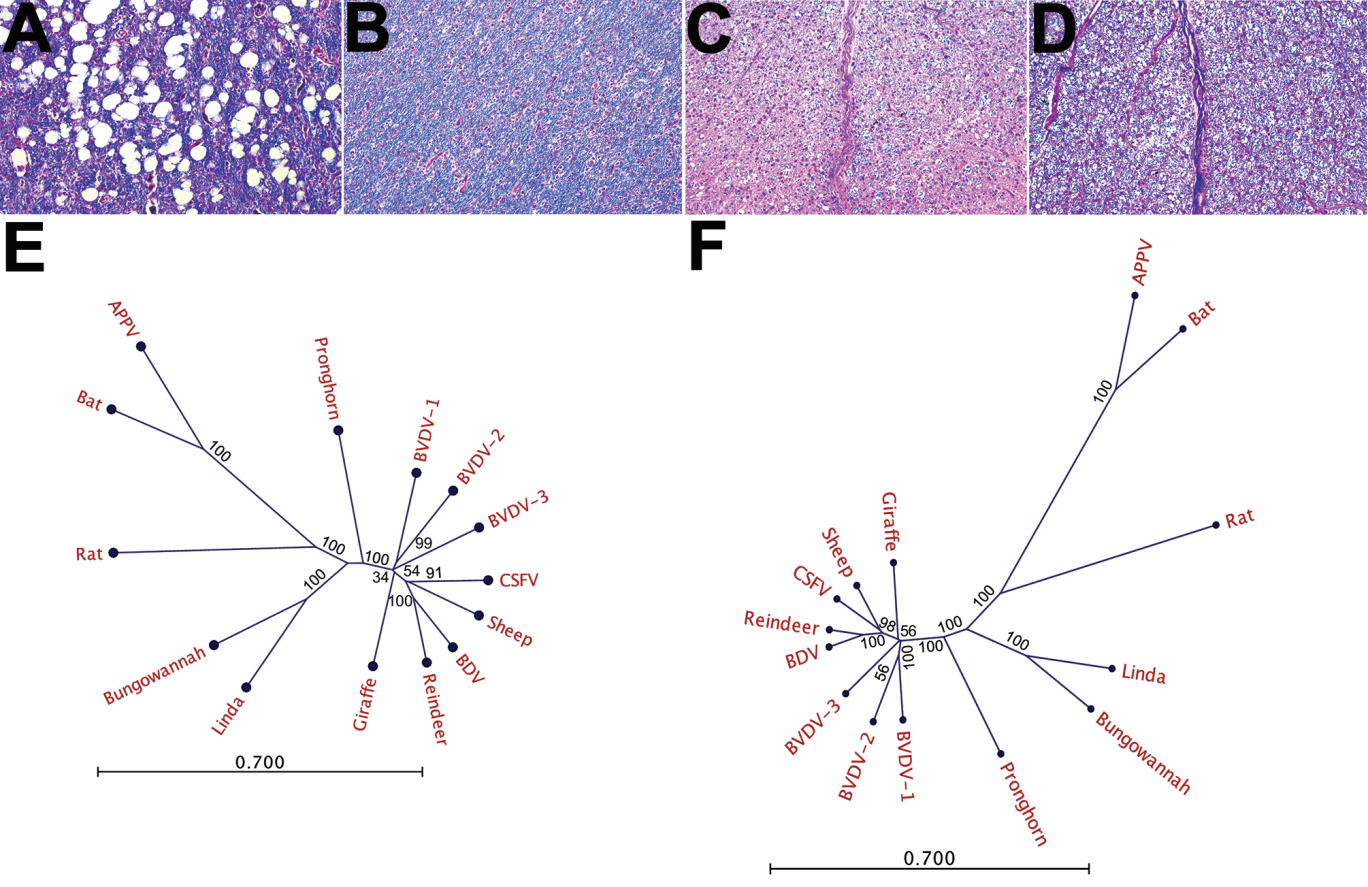
Histologic and phylogenetic examination in investigation of piglets with congenital tremor (CT) on a farm in southeastern Austria, 2015. A) Cerebellar white matter of CT-affected piglet showing multiple sharply bordered vacuoles but normal myelination (stained in blue; luxol fast blue/hematoxylin-eosin staining; original magnification ×10). B) Control piglet with normal cerebellar white matter (original magnification ×10). C) Spinal cord white matter in CT-affected piglet shows a severely reduced amount of myelin (original magnification ×10). D) Control piglet with normal myelination of the spinal cord white matter (original magnification ×10). E, F) Phylogenetic neighbor-joining analysis using the nucleotide (E) and polyprotein (F) sequences of the novel virus isolated from piglets, provisionally termed Linda virus (GenBank accession no. KY436034); approved pestivirus species BVDV-1 NADL (accession no. M31182.1), BVDV-2 890 (accession no. U18059.1), CSFV Alfort_187 (accession no. X87939.1), and BDV X818 (accession no. AF037405.1); and tentative species Bungowannah virus (accession no. EF100713.2), sheep pestivirus Aydin (accession no. NC_018713.1), pronghorn pestivirus (accession no. NC_024018.2), reindeer pestivirus (accession no. AF144618.2), giraffe pestivirus (accession no. NC_003678.1), BVDV-3 D32_00_HoBi (accession no. AB871953.1), APPV NL1 (accession no. KX929062.1), *Rhinolophus affinis* pestivirus (accession no. JQ814854.1), and Norway rat pestivirus (accession no. KJ950914.1). Assumed relationships between the species are shown in a radial branching diagram with numbers indicating the bootstrap values of 1,000 replicates in percentages. Scale bars indicate number of substitutions per site. BDV, border disease virus; BVDV, bovine viral diarrhea virus; CSFV, classical swine fever virus.

A small-scale piglet-producing farm in Styria, in southeastern Austria, reported major piglet losses from CT in January 2015. Animals from this farm were examined and samples were taken by authorized veterinarians. The CT-affected piglets showed a severe lateral shaking and were often incapable of sucking milk, which led to elevated preweaning death rates (Video. The prevalence of CT varied from 20% to 100% within the affected litters. Litters affected by CT were not used for the production of replacement gilts. The outbreak of CT abruptly stopped in July 2015, when all sows had produced 1 CT-affected litter. Only 22.4 piglets per sow were weaned in that year, compared with an average of 25.8 piglets/sow/year before CT symptoms occurred. Pathological examinations were performed as described previously (*8*), confirming the presence of typical CT A-II lesions (Figure 1, panel A).

**Video:**
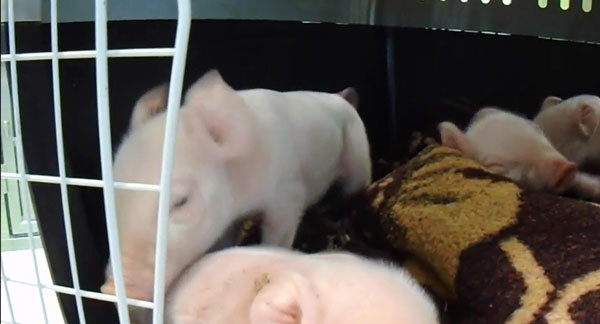
Congenital tremor visible in piglets that were affected by a novel pestivirus provisionally termed Linda virus, Austria, 2015.

We tested various samples from the farm using an APPV-specific TaqMan probe-based reverse transcription PCR (RT-PCR) (*8*) but obtained negative results. However, we obtained an amplicon of appropriate length from CT-piglet serum samples by using a novel panpestivirus RT-PCR (PPF 5′-GTKATHCAATACCCTGARGC-3′ and PPR 5′-GGRTTCCAGGARTACATCA-3′), which enables detection of CSFV, BVDV-1, BVDV-2, BDV, BV, and APPV. Surprisingly, sequencing of this RT-PCR product yielded an unknown sequence with a noninterrupted open reading frame. An initial BLAST search (https://blast.ncbi.nlm.nih.gov/Blast.cgi) resulted in “no significant similarity found,” but the translated sequence of 270 aa aligned well with different pestiviruses. The viral RNA was detected in all samples (serum, tonsils, lung, liver, spleen, and central nervous system material) of CT-affected piglets and their littermates from the farm.

After inoculating SK-6 cells with serum samples of affected piglets, we detected the amplification of this unknown pestivirus using RT-PCR. We provisionally termed the agent “Linda” (lateral-shaking inducing neurodegenerative agent) to avoid confusion with other pestiviruses. We determined the full genome of Linda virus (LV) using the standard primer walking RT-PCR approach together with rapid amplification of cRNA ends PCR to identify the ultimate 5′- and 3′- termini using the cultured virus. We determined the length of the LV genome (GenBank accession no. KY436034) to be 12,614 nt, with 381 nt 5′-nontranslated region, an open reading frame of 11,772 nt, and 461 nt of 3′-nontranslated region. 

We performed a phylogenetic analysis of LV using CLC Workbench 7.6 (CLCBIO, Aarhus, Denmark), which demonstrated LV’s divergence from other pestiviruses (Figure 1, panel B). We found the identity between LV, approved pestiviruses, and APPV to be only 60%, but we found an identity of 68% between LV and BV. Comparison of the pestiviral polyprotein sequences yielded an amino acid identity of 69% between LV and BV and of <54% with all other pestiviruses (Figure 1, panel C). All known cleavage sites of the NS3 protease were present in LV (*10*). We mapped the NS4A-NS4B cleavage, which takes place at an L/A site in classical pestiviruses and at an L/S site in BV, to the L_2354_/S_2355_ motive in LV; the intramolecular NS3 cleavage site (L_1834_/A_1835_) was conserved in LV (*11*). Each hypothetical mature protein of LV matched best with the mature proteins of BV (Table), even if the identities were relatively low in the structural protein region.

**Table:** BLAST analysis of the 12 putative mature proteins and the polyprotein of novel pestivirus Linda virus from pigs with congenital tremor, Austria, 2015*

Putative mature protein	Amino acid region	Best BLAST hit (GenBank accession no.)	Alignment coverage, %	E-value	Identity, %
N^pro^	1–182	BV (YP_008992092.1)	96	4 × 10^–64^	60
Core	183–283	BV (YP_008992092.1)	95	3 × 10^–43^	83
E^rns^	284–504	BV (YP_008992092.1)	100	5 × 10^–117^	74
E1	505–702	BV (YP_008992092.1)	100	3 × 10^–82^	67
E2	703–1077	BV (YP_008992092.1)	98	4 × 10^–140^	53
P7	1078–1152	BV (YP_008992092.1)	100	1 × 10^–25^	60
NS2	1153–1608	BV (YP_008992092.1)	99	0.0	63
NS3	1609–2291	BV (YP_008992092.1)	100	0.0	85
NS4A	2292–2354	BV (YP_008992092.1)	100	2 × 10^–28^	81
NS4B	2355–2701	BV (YP_008992092.1)	100	0.0	78
NS5A	2702–3205	BV (YP_008992092.1)	100	0.0	53
NS5B	3206–3923	BV (YP_008992092.1)	99	0.0	73

*BV, Bungowannah virus; E, envelope; NS, nonstructural.

**Figure 2:**
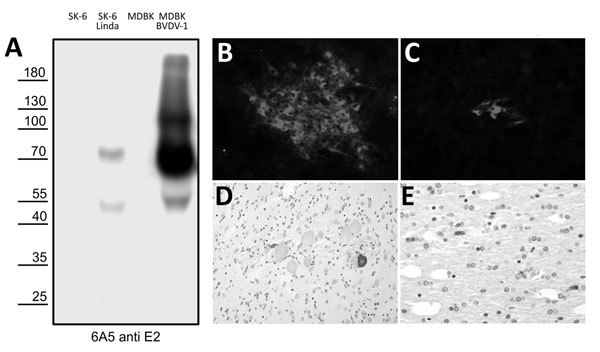
Detection of pestivirus E2 protein with monoclonal antibody 6A5 in investigation of piglets with congenital tremor (CT) on a farm in southeastern Austria, 2015. A) Western blot analysis of cells infected with novel virus provisionally termed Linda virus. Total protein of SK-6 cells infected with Linda virus and MDBK cells infected with BVDV-1 (strain NADL) was probed with the pestivirus E2-specific antibody 6A5. The apparent molecular mass of monomeric E2 (LV 50 kDa and BVDV-1 55 kDa) shows that Linda virus E2 has a lower molecular weight than BVDV-1 E2 as a result of fewer N-linked glycosylation sites. In contrast, the mass of E1-E2 heterodimers is comparable (≈70 kDa in Linda virus and BVDV-1), because Linda virus E1 has an additional N-linked glycosylation site. B, C) Focus size of Linda virus 48 hours after infection of SK-6 cells (B) and MDBK cells (C) (original magnification ×20). D, E) Detection of pestiviral E2 within neuronal tissue of Linda virus–positive, congenital tremor–affected piglets showing positive signals in neurons of the nucleus of the trigeminal nerve (D) (original magnification ×10) and within glial cells in the cerebellar white matter (E) (original magnification ×20). BVDV, bovine viral diarrhea virus.

We saw a strong reactivity against LV infected cells when using a BVDV E2-specific antibody termed 6A5 (*12*). This antibody detected the E1-E2 heterodimer of LV (75 kDa) as well as the E2 monomer (50 kDa) in Western blot analysis, although the reactivity against BVDV-1 E2 was much stronger (Figure 2, panel A). With the help of this antibody, we were able to study the in vitro growth of LV. E2 antigen signals of LV were detectable in the cytoplasm of SK-6 and MDBK cells after infection, but the antigen-positive foci were 10-fold larger in SK-6 than in MDBK cells (Figure 2, panels B,C). Already in the primary passage on porcine cells, considerably high infectious titers of LV were measured in the supernatant (>10^7^ 50% tissue culture infectious dose/mL). Using monoclonal antibody 6A5 in immunohistochemical analysis, we detected pestiviral antigen in CT-affected piglets in regions with histological lesions. Specific stains in neurons of the nucleus of the trigeminal nerve (Figure 2, panels D,E), in glial cells in the cerebellar and the cerebral white matter, and in tubular epithelium of the kidneys proved that a pestivirus crossed the blood–brain barrier. As in the case of other pestiviruses, the mechanisms inducing hypomyelination during fetal development remain unclear (*8*,*13*).

## Conclusions

A previously unknown pestiviral agent was found in CT-affected piglets on a farm in Austria. We observed a severe hypomyelination in the entire white matter of the spinal cord and detected pestivirus antigen in the brain of the CT-affected piglets, suggesting a causal relationship between infection and lesions. Analyses of the assembled genome allowed an unambiguous assignment of LV within the genus *Pestivirus* with regard to the presence of pestivirus-specific genes (N^pro^ and E^rns^) and sequence homology to other pestiviruses (*14*). In contrast to APPV, which hardly infects cultured cells at all, LV could be easily propagated on porcine cell lines without the need for adaptation, similar to what has been reported for BV. We suggest that LV likely shares a common ancestor with BV.

After its description ≈10 years ago, BV was intensively sought worldwide, but the virus has never been detected outside Australia. The broad cell culture tropism of BV led to the hypothesis that the virus recently jumped from another species to porcine hosts (*15*). The discovery of a related pestivirus with substantial sequence divergence in swine on a different continent suggests that both viruses probably have a porcine origin. The identification of the cross-reacting E2-specific monoclonal antibody 6A5 indicates that a cross-reactivity to related pestiviral proteins exists, which might interfere with the serologic testing for CSFV. Further work is needed to investigate the prevalence and the epidemiology of LV in Europe and to assess its virulence in controlled animal experiments.

## References

[1] King AMQ, Adams MJ, Carstens EB, Lefkowitz EJ; International Union of Microbiological Societies, Virology Division. Virus taxonomy: classification and nomenclature of viruses. Ninth report of the International Committee on Taxonomy of Viruses. Amsterdam: Elsevier/Academic Press; 2012.

[2] Kirkland PD, Frost MJ, Finlaison DS, King KR, Ridpath JF, Gu X. Identification of a novel virus in pigs—Bungowannah virus: a possible new species of pestivirus. Virus Res. 2007;129:26–34. 10.1016/j.virusres.2007.05.002 17561301

[3] Hause BM, Collin EA, Peddireddi L, Yuan F, Chen Z, Hesse RA, et al. Discovery of a novel putative atypical porcine pestivirus in pigs in the USA. J Gen Virol. 2015;96:2994–8. 10.1099/jgv.0.000251 26219947

[4] Arruda BL, Arruda PH, Magstadt DR, Schwartz KJ, Dohlman T, Schleining JA, et al. Identification of a divergent lineage porcine pestivirus in nursing piglets with congenital tremors and reproduction of disease following experimental inoculation. PLoS One. 2016;11:e0150104. 10.1371/journal.pone.0150104 26909691 PMC4766193

[5] Postel A, Hansmann F, Baechlein C, Fischer N, Alawi M, Grundhoff A, et al. Presence of atypical porcine pestivirus (APPV) genomes in newborn piglets correlates with congenital tremor. Sci Rep. 2016;6:27735. 10.1038/srep27735 27292119 PMC4904412

[6] Beer M, Wernike K, Dräger C, Höper D, Pohlmann A, Bergermann C, et al. High prevalence of highly variable atypical porcine pestiviruses found in Germany. Transbound Emerg Dis. 2016. 10.1111/tbed.12532 27297961

[7] de Groof A, Deijs M, Guelen L, van Grinsven L, van Os-Galdos L, Vogels W, et al. Atypical porcine pestivirus: a possible cause of congenital tremor type A-II in newborn piglets. Viruses. 2016;8:271. 10.3390/v8100271 27782037 PMC5086607

[8] Schwarz L, Riedel C, Högler S, Sinn LJ, Voglmayr T, Wöchtl B, et al. Congenital infection with atypical porcine pestivirus (APPV) is associated with disease and viral persistence. Vet Res (Faisalabad). 2017;48:1. 10.1186/s13567-016-0406-1 28057061 PMC5217315

[9] Barlow RM. Morphogenesis of hydranencephaly and other intracranial malformations in progeny of pregnant ewes infected with pestiviruses. J Comp Pathol. 1980;90:87–98. 10.1016/0021-9975(80)90031-6 6248579

[10] Lamp B, Riedel C, Roman-Sosa G, Heimann M, Jacobi S, Becher P, et al. Biosynthesis of classical swine fever virus nonstructural proteins. J Virol. 2011;85:3607–20. 10.1128/JVI.02206-10 21270154 PMC3067844

[11] Lamp B, Riedel C, Wentz E, Tortorici MA, Rümenapf T. Autocatalytic cleavage within classical swine fever virus NS3 leads to a functional separation of protease and helicase. J Virol. 2013;87:11872–83. 10.1128/JVI.00754-13 23986594 PMC3807365

[12] Gilmartin AA, Lamp B, Rümenapf T, Persson MA, Rey FA, Krey T. High-level secretion of recombinant monomeric murine and human single-chain Fv antibodies from *Drosophila* S2 cells. Protein Eng Des Sel. 2012;25:59–66. 10.1093/protein/gzr058 22160929 PMC3258843

[13] Bradley R, Done JT, Hebert CN, Overby E, Askaa J, Basse A, et al. Congenital tremor type AI: light and electron microscopical observations on the spinal cords of affected piglets. J Comp Pathol. 1983;93:43–59. 10.1016/0021-9975(83)90042-7 6841693

[14] Liu L, Xia H, Wahlberg N, Belák S, Baule C. Phylogeny, classification and evolutionary insights into pestiviruses. Virology. 2009;385:351–7. 10.1016/j.virol.2008.12.004 19167739

[15] Kirkland PD, Read AJ, Frost MJ, Finlaison DS. Bungowannah virus—a probable new species of pestivirus—what have we found in the last 10 years? Anim Health Res Rev. 2015;16:60–3. 10.1017/S1466252315000031 26050573

